# The Effect of a Combination Treatment Using Palonosetron, Promethazine, and Dexamethasone on the Prophylaxis of Postoperative Nausea and Vomiting and QTc Interval Duration in Patients Undergoing Craniotomy under General Anesthesia: A Pilot Study

**DOI:** 10.3389/fmed.2016.00001

**Published:** 2016-02-02

**Authors:** Sergio D. Bergese, Erika G. Puente, Maria A. Antor, Gerardo Capo, Vedat O. Yildiz, Alberto A. Uribe

**Affiliations:** ^1^Department of Anesthesiology, The Ohio State University Medical Center, Columbus, OH, USA; ^2^Department of Neurological Surgery, The Ohio State University Medical Center, Columbus, OH, USA; ^3^Department of Anesthesiology, Jackson Memorial Hospital, University of Miami, Miami, FL, USA; ^4^College of Arts and Sciences, The Ohio State University, Columbus, OH, USA; ^5^Center for Biostatistics, The Ohio State University, Columbus, OH, USA

**Keywords:** nausea, vomiting, emesis, postoperative complications, QTc

## Abstract

**Introduction:**

Postoperative nausea and vomiting (PONV) is a displeasing experience that distresses surgical patients during the first 24 h after a surgical procedure. The incidence of postoperative nausea occurs in about 50%, the incidence of postoperative vomiting is about 30%, and in high-risk patients, the PONV rate could be as high as 80%. Therefore, the study design of this single arm, non-randomized, pilot study assessed the efficacy and safety profile of a triple therapy combination with palonosetron, dexamethasone, and promethazine to prevent PONV in patients undergoing craniotomies under general anesthesia.

**Methods:**

The research protocol was approved by the institutional review board and 40 subjects were provided written informed consent. At induction of anesthesia, a triple therapy of palonosetron 0.075 mg IV, dexamethasone 10 mg IV, and promethazine 25 mg IV was given as PONV prophylaxis. After surgery, subjects were transferred to the surgical intensive care unit or post anesthesia care unit as clinically indicated. Ondansetron 4 mg IV was administered as primary rescue medication to subjects with PONV symptoms. PONV was assessed and collected every 24 h for 5 days via direct interview and/or medical charts review.

**Results:**

The overall incidence of PONV during the first 24 h after surgery was 30% (*n* = 12). The incidence of nausea and emesis 24 h after surgery was 30% (*n* = 12) and 7.5% (*n* = 3), respectively. The mean time to first emetic episode, first rescue, and first significant nausea was 31.3 (±33.6), 15.1 (±25.8), and 21.1 (±25.4) hours, respectively. The overall incidence of nausea and vomiting after 24–120 h period after surgery was 30% (*n* = 12). The percentage of subjects without emesis episodes over 24–120 h postoperatively was 70% (*n* = 28). No subjects presented a prolonged QTc interval ≥500 ms before and/or after surgery.

**Conclusion:**

Our data demonstrated that this triple therapy regimen may be an adequate alternative regimen for the treatment of PONV in patients undergoing neurological surgery under general anesthesia. More studies with a control group should be performed to demonstrate the efficacy of this regimen and that palonosetron is a low risk for QTc prolongation.

**ClinicalTrials.gov Identifier:**

NCT02635828 (https://clinicaltrials.gov/show/NCT02635828).

## Introduction

Postoperative nausea and vomiting (PONV) is a displeasing experience that distresses surgical patients during the first 24 h after a surgical procedure (1–8). The incidence of postoperative nausea in general population occurs in about 50%, the incidence of postoperative vomiting is about 30%, and in high-risk patients, the PONV rate could be as high as 80% (2, 3, 5, 6, 8–10). In addition, the literature reports a PONV incidence after craniotomy of 43–70% (10, 11). PONV could be a moderate-to-severe postoperative problem that increases the chances of developing several complications, including suture dehiscence, bronchopulmonary aspiration, elevated intraocular and intracranial pressures, delay recovery and discharge time, etc. (2, 4, 7, 9, 12).

According to the Consensus Guidelines for the Management of Postoperative Nausea and Vomiting, a combination of antiemetic medications is recommended as the most appropriate regimen for patients with moderate and high-risk factors for PONV (6). Early PONV are those episodes that occur in a 0–2 h timeframe, while delayed PONV occurs during the 2–24 h period (6). Postdischarge nausea and vomiting (PDNV) are episodes that occur after the patient has left the hospital (6). PONV is a complex occurrence that can be triggered by numerous receptor pathways [dopamine type 2, serotonin type 3 (5-HT3), histamine type 1, muscarinic cholinergic type 1, and neurokinin type 1]; therefore, we have several alternatives to prevent and treat PONV (2, 3, 5, 6, 8).

The Apfel Simplified Risk Score is one of the two most commonly used and effective risk scores to assess inpatient risk of developing PONV when undergoing inhaled anesthesia. The Apfel Simplified Risk Score is based on four predictors: female gender, non-smoking status, history of PONV and/or motion sickness, and use of postoperative opioids. The incidence of PONV with the presence of 0, 1, 2, 3, and 4 risk factors increases drastically, by 10, 20, 40, 60, and 80%, respectively. Patients with 0–1 are considered low risk, those patients with 2 risk factors fall into “medium risk”; and those patients with 3 or more risk factors are considered to be “high risk” (6).

Literature describes dexamethasone as an effective drug to prevent PONV (6–8, 13). These data show it to be an inexpensive and excellent antiemetic prophylactic agent commonly used worldwide, especially if it is administrated early before surgery (6–8, 13). Its mechanism of action remains undetermined; most likely, its peripherally prostaglandin antagonism, serotonergic antagonism, and endorphin releasing properties play important roles in PONV management. It has a long biological half-life of 36–48 h and excellent side-effects profile after a single dose of 8 mg intravenous (IV) given before anesthesia induction. However, the literature reported cases with awake patients experiencing perineal burning, itching, and tingling after receiving a dose of dexamethasone 8 mg IV for PONV prophylaxis (14).

Promethazine is a phenothiazine derivative that competitively blocks histamine [H(1)] receptors and exhibits antiemetic and sedative properties. IV infusion of promethazine typically relieves PONV after 5 min and lasts for 2–6 h (7, 15). However, in ambulatory surgical settings, promethazine has shown concerning sedative effects (7, 8, 15).

Palonosetron is the latest serotonin receptor antagonist (3, 6–8, 13, 16, 17). Serotonin is an ubiquitous central and peripheral neurotransmitter thought to be the predominant mediator of the perception of nausea and triggering of the vomiting response (18). This occurs in both the brain and the periphery via the serotonin 5-hydroxytryptamine type 3 [5-HT(3)] receptor pathways (19). Palonosetron, compared to older 5-HT3 receptor antagonist, such as ondansetron, granisetron, dolasetron, and ramosetron, has higher receptor binding affinity and longer plasma half-life (40 h) (3, 6–8, 13, 16, 17). The most effective dose for PONV is a single 0.075 mg IV dose administered over 10 s right before anesthesia induction (5, 6, 12, 13). The most common side effects of palonosetron described in the literature are electrocardiogram QT prolongation, bradycardia, headache, and constipation (18, 20). The US Food and Drug Administration (FDA) approved its use for prevention of PONV in March 2008 (7, 20). Palonosetron is a fairly new 5-HT3 receptor antagonist, and studies have shown controversial results concerning QTc prolongation (5, 16).

QTc prolongation may suggest an inherited predisposition to sudden cardiac death. It may also uncover an increased vulnerability to the development of life threatening ventricular tachycardia in patients being considered for treatment with a known QT-prolonging drug (21–24). The American Heart Association/American College of Cardiology Foundation (AHA/ACCF) describes a QTc of >470 ms for males and >480 ms for females as prolonged, with a QTc value >500 ms considered highly abnormal for both genders (22, 23, 25). Consequently, the literature shows data with direct relationship between palonosetron administration and QTc prolongation (26). Nonetheless, other studies have shown palonosetron to have none to mild effects on the QTc interval (5, 16).

Therefore, the study design of this single arm, non-­randomized, pilot study assessed the efficacy and safety profile of a triple therapy combination with palonosetron, dexamethasone, and promethazine to prevent PONV in patients undergoing craniotomies under general anesthesia.

## Methods

The research protocol was approved by the institutional review board and 40 subjects provided written informed consent prior to any research procedures at The Ohio State University Wexner Medical Center and completed the study. The protocol was not registered in clinicaltrial.gov during enrollment period because it was not part of our institutional standard operation procedures; thus, it was registered after enrollment completion (ClinicalTrials.gov Identifier: NCT02635828). The study’s inclusion criteria consisted of adult patients, 18–85 years, undergoing neurological surgery (opening of the cranium and dura mater) requiring at least 1 h of general anesthesia and at least 72 h of postoperative hospitalization. These subjects were assessed preoperatively using the Apfel Simplified Risk Score. Exclusion criteria consisted of subject with prisoner or mentally ill status, history of alcohol or drug abuse, hypersensitivity to any of the medications administered in the protocol, female subjects who are pregnant or breastfeeding, history of vomiting or severe nausea 24 h prior to a procedure, history of treatment with any drug or treatment within last 24 h prior to start of the treatment, and/or subjects who have participated in any other clinical study.

An electrocardiogram (ECG) was obtained at baseline, 24 and 120 h after surgery, if the subject remained hospitalized. At induction of anesthesia, a triple therapy of palonosetron 0.075 mg IV, dexamethasone 10 mg IV, and promethazine 25 mg IV was given as PONV prophylaxis.

The general anesthesia regimen recommended began with premedication of midazolam 1–2 mg IV promptly before the subject was transferred to the operating room. Propofol 1–2 mg/kg IV and fentanyl 0.75–1.5 μg/kg IV were used to induce anesthesia. Rocuronium 0.6–1.2 mg/kg^−1^ was administered before tracheal intubation. General anesthesia was preserved with volatile anesthetics (sevoflurane, desflurane, or isoflurane) and clinical judgment regulated titration concentrations. The analgesia during anesthesia maintenance was done with Fentanyl boluses of 0.5–2.0 μg/kg^−1^ IV and/or hydromorphone boluses 0.5 mg IV. In order to reverse the residual neuromuscular block at the end of the procedure, neostigmine and glycopyrrolate were used.

After surgery, subjects were transferred to the surgical intensive care unit (SICU) or post anesthesia care unit (PACU) as clinically indicated. Ondansetron 4 mg IV was administered as primary rescue medication to subjects with PONV symptoms. Subjects who did not respond to this initial treatment were given a second line of therapy. PONV was assessed and collected every 24 h for 5 days via direct interview and/or medical charts review. Episodes of nausea, vomiting, and administration of rescue therapy for either nausea or vomiting were recorded, in addition to the severity of each nauseous or emetic episode. Nausea was rated by the patients utilizing a verbal response scale (0–10), with 0 being no nausea at all and 10 being severe nausea. Vomiting was evaluated by the investigator or nursing staff numerically as either 0 (no vomiting), 1 (mild vomiting), 2 (moderate vomiting), or 3 (severe vomiting). Following the first 24 h after administration of the prophylactic triple therapy, an ECG was conducted and standard of care blood was drawn for safety analysis. If the patient was discharged before the 5-day time period, the patient was then contacted via phone call by research personnel to complete PONV and safety assessments.

Descriptive statistics are reported as mean ± SE and median (range) or total number and percentage. Binomial test were performed to compare the Apfel risk factors between our study and literature. These data were analyzed using Statistical Analysis Software, version 9.3 (SAS Institute Inc., Cary, NC, USA). Significance was accepted if *p* ≤ 0.05.

## Results

**Figure 1:**
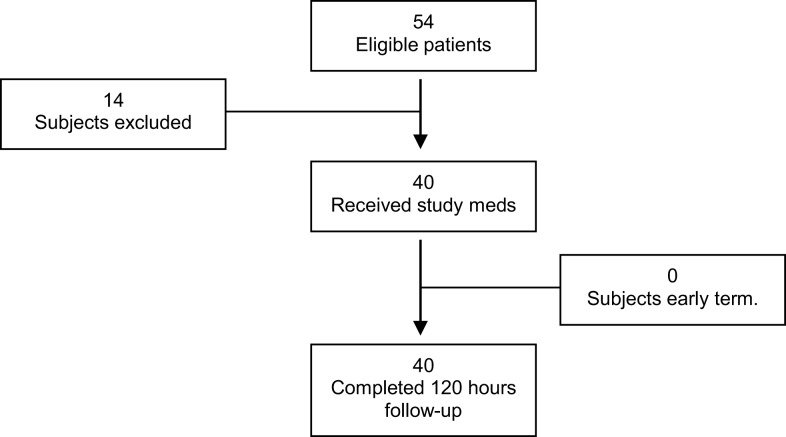
**Patient screening flowchart**.

A total of 54 subjects were enrolled and screened for entry into the study. Of the 54 subjects who were screened, 14 were screen failures and 40 subjects were eligible to complete the study through 120 h follow-up. The primary reasons for screen failure were failure to meet all the inclusion/exclusion criteria (*n* = 5), consent withdrawal (*n* = 0), and others (*n* = 9) (Figure 1). No side effects associated with study medications showed significant differences. The subject’s demographics, surgical variables, and Apfel risk factors are listed in Table 1.

**Table 1:** **Patient demographics and surgical variables**.

Demographics and surgical variables	Palonosetron
Number of subjects	40
Age, mean (SD), years	50.6 (16.7)
Weight, mean (SD), kg	84.0 (18.4)
Height, mean (SD), cm	170.4 (9.8)
BMI, mean (SD)	28.8 (5.8)
Race-White, *n* (%)	39 (97)
ASA I/II/III	0/8/32
Female, *n* (%)	21 (52)
History of PONV and/or motion of sickness *n* (%)	6 (15)
Non-smoker status, *n* (%)	32 (80)
Postoperative opioids, *n* (%)	40 (100)
Apfel risk factors, *n* (%)	
1 (low risk)	2 (5%)
2 (moderate risk)	21 (52.5%)
3 or 4 (high risk)	17 (42.5%)
Duration of anesthesia, mean (SD), h	4.5 (2.0)
Duration of SICU stay, mean (SD), h	54.9 (43.5)
Duration of total hospital stay, mean (SD), h	66.2 (45.3)

*IQR, interquartile range*.

The overall incidence of PONV during the first 24 h after surgery was 30% (*n* = 12). The incidence of nausea and emesis 24 h after surgery was 30% (*n* = 12) and 7.5% (*n* = 3), respectively. Of those patients who experienced nausea during the first 24 h after surgery, the median worst nausea score was 6 (8, 10). The median severity of vomiting was 2 (1, 3), corresponding to moderate severity. Rescue medication during the first 24 h was used on 12 patients (30%) (Table 2).

**Table 2:** **Postoperative nausea and vomiting outcome variables in the first 24 h**.

PONV outcome variables	Palonosetron
Number of subjects	40
PONV, *n* (%)	12 (30%)
Vomiting, *n* (%)	3 (7.5%)
Worst vomiting score, median (IQR)	2 (1, 3)
Any nausea incidence, *n* (%)	12 (30%)
Significant Nausea incidence (a score ≥4 on the VRS), *n* (%)	11 (27.5%)
Worst nausea score, median (IQR)	6 (8, 10)
Rescue antiemetics, *n* (%)	12 (30%)
Postoperative opioid consumption, median (IQR), oral morphine, mg	68.5 (36, 140)

*VRS = verbal rating scale, IQR = interquartile range*.

The mean time to first emetic episode, first rescue, and first significant nausea was 31.3 (±33.6), 15.1 (±25.8), and 21.1 (±25.4) hours, respectively (Table 3).

**Table 3:** **Intent to treat population**.

Time to treatment failure, mean (SD)	Palonosetron
Number of subjects	40
Time to first emetic episode	31.3 (33.6)
Time to first rescue	15.1 (25.8)
Time to first significant nausea	21.1 (25.4)

In the course of the early PONV (0–2 h), the incidence of PONV was 10% (*n* = 4), nausea and vomiting occurred in 10% (*n* = 4) and 0% (*n* = 0), respectively. The worst nausea and vomiting score was 9 (8, 10) and 3 (3, 3), respectively (Table 4).

**Table 4:** **Postoperative nausea and vomiting outcome variables in the first 24 h**.

Outcome variables	0–2 h Palonosetron	24–48 h Palonosetron	24–72 h Palonosetron	24–96 h Palonosetron	24–120 h Palonosetron
Number of subjects	40	40	40	40	40
PONV, *n* (%)	4 (10)	9 (22.5)	11 (27.5)	12 (30)	12 (30)
Vomiting, *n* (%)	0	2 (5)	4 (10)	5 (12.5)	6 (15)
Worst vomiting score, median (IQR)	3 (3, 3)	3 (3, 3)	2.5 (1.5, 3)	2 (2, 3)	2 (2, 3)
Any nausea incidence, *n* (%)	4 (10)	9 (22.5)	19 (47.5)	25 (62.5)	27 (67.5)
Significant nausea incidence (a score ≥4 on the VRS), *n* (%)	4 (10)	8 (20)	17 (42.5)	23 (57.5)	25 (62.5)
Worst nausea score, median (IQR)	9 (8, 10)	7 (6, 10)	6 (5, 10)	6 (6, 10)	6 (6, 10)
Rescue antiemetics, *n* (%)	4 (10)	2 (5)	2 (5)	2 (5)	2 (5)
Postoperative opioid consumption, median (IQR), oral morphine milligram	NA	45 (27, 117.5)	45 (27, 98.7)	45 (30, 91)	45 (30, 90)

*VRS = verbal rating scale; IQR = interquartile range*.

The overall incidence of nausea and vomiting after 24–120 h period after surgery was 30% (*n* = 12). The percentage of subjects without emesis episodes over 24–120 h postoperatively was 70% (*n* = 28). Of those subjects who experienced nausea during 24–120 h after surgery, the median severity was 6 (6.0, 10). The median severity of vomiting was 2.5 (2, 3), corresponding to moderate severity (Table 4).

No subjects presented a prolonged QTc interval ≥500 ms before and/or after surgery. The percentage of subjects who presented a prolonged QTc interval ≥450 ms at baseline and 24 h after surgery were 22.5% (*n* = 9) and 13.9% (*n* = 5), respectively. The incidence of a change in QTc interval larger than 60 ms 24 h after surgery was 2.8% (*n* = 1). The incidence of prolonged QTc interval ≥450 ms 120 h post-surgery was 19.2% (*n* = 5). The percentage of subjects who presented a change in QTc interval larger than 30 and 60 ms were 11.5% (*n* = 3) and 0%, respectively (Table 5).

**Table 5:** **QTc variables**.

	Baseline *N* (40)	PO 24 h *N* (36)	PO 120 h *N* (26)
Prolonged QTc interval >500 ms	0 (0%)	0 (0%)	0 (0%)
Prolonged QTc interval >450 ms	9 (22.5%)	5 (13.9%)	5 (19.2%)
δQTc interval >30 ms	NA	1 (2.8%)	3 (11.5%)
δQTc interval >60 ms	NA	1 (2.8%)	0 (0%)

## Discussion

This study successfully demonstrates that triple therapy with palonosetron, dexamethasone, and promethazine for prevention of postoperative nausea and vomiting in patients undergoing neurological surgery and general anesthesia is an adequate alternative. It has been established that the uncomfortable effects of vomiting on intracranial pressure can lead to permanent and catastrophic consequences on patients undergoing craniotomy (10).

A PONV incidence of 40–80% is predicted by the Apfel scale in patients with medium to high risk (6). Nonetheless, our study reported a PONV incidence of 24–46% (Table 6) having statistical significance results in subjects with four risk factors with a PONV incidence of 25% compared with 80% described by Gan et al. (6). These facts support our hypothesis that triple therapy with palonosetron, dexamethasone, and promethazine does indeed reduce the incidence of PONV in subjects undergoing craniotomy and can be used as an alternative regimen.

**Table 6:** **Apfel comparison in PONV 24 h: literature vs. our findings**.

Apfel risk factor	*N* **=** 40	PONV 24 h incidence	Risk for PONV (Apfel) (%)	*p*-Value	Difference CI
1	2	0 (0.0%)	20	NA	NA
2	21	5 (23.9%)	40	0.132	(0.05, 0.42)
3	13	6 (46.2%)	60	0.309	(0.19, 0.73)
4	4	1 (25%)	80	0.006	(−0.17, 0.67)

The most common side effects that could be associated with palonosetron described in clinical trials are ECG QT prolongation (5%), bradycardia (4%), headache (3%), and constipation (2%) (20). The results from our study showed only a 2.8% of ECG QT prolongation >60 ms and none of the subjects had ECG QT prolongation >500 ms. The rest of common side effects were challenging to assess in neurosurgical settings.

In our study, only 13.9% of patients presented a prolonged QTc interval of ≥450 ms and 0% of patients presented a prolonged QTc interval ≥500 ms. Since prolongation of QTc interval was not our primary objective, more studies with a proper power analysis should be performed to conclude that palonosetron is a low risk for QTc prolongation.

Latz et al. presented a prospective study that evaluated the incidence and risk factors of PONV in 229 patients undergoing craniotomy (10). It found a PONV incidence in 47% of patients (10). In our study, palonosetron was demonstrated to be an effective antiemetic on the triple therapy by reducing the PONV incidence to 30%.

Chun et al. published a randomized, double-blind, placebo-controlled trial that proved the efficacy of palonosetron as PONV prophylaxis in 204 patients (27). In this study, the palonosetron group presented 33% incidence of PONV compared to 47% of the placebo group on a 0–24 h postoperative period (27). In addition, the PONV incidence in the 0–72 h period was 33% for palonosetron compared to 53% for the placebo group (27). Our study showed a 30% PONV incidence that shows a strong correlation with this study.

Mansour designed a double-blind, active controlled study with 150 patients comparing the use of dexamethasone alone, dexamethasone–metoclopramide therapy, or dexamethasone–palonosetron combination therapy (28). The dexamethasone–palonosetron combination therapy proved to be the most effective by significantly reducing the PONV incidence 12–24 h after surgery compared with dexamethasone and dexamethasone–metoclopramide therapies (16 vs. 48 vs. 40%, respectively) (28).

Min et al. presented a retrospective study that analyzed a database of 81 patients who underwent general anesthesia. The main focus of this study was to evaluate changes in QTc interval throughout different time points in surgery (incision, 30, 60, 90, and 120 min after incision). The results demonstrated that the incidence of QTc prolongation ≥500 ms was higher in patients who received 0.075 mg of palonosetron intravenously than those who did not during general anesthesia (26). Nonetheless, this study had several limitations, including not being a randomized prospective study (26). Therefore, factors that could have affected the QTc interval were not controlled (26). On the other hand, in our study, there were no clinically important changes in the baseline, 24 h period and discharge ECG, and no shifts of clinical concerns noted.

A study by Morganroth et al. included 221 healthy subjects who were assigned to receive one of the following five treatments: placebo, palonosetron (0.25, 0.75, or 2.25 mg), or moxifloxacin (400 mg) in order to study the effects of palonosetron on QTc interval (29). All three doses of palonosetron showed no significant effects on QTc prolongation (29). The main objective of the study was developed in accordance with the International Conference on Harmonization of Technical Requirements for Registration of Pharmaceuticals for Human Use (ICH) E14 in order to analyze the potential of cardiovascular toxicity (29). No significant QTc prolongations were detected (29). This supports our results for QTc prolongation.

Our study also presented certain limitations that should be mentioned. To begin with, this study enrolled 40 subjects who received study medication and completed all the assessments, but the study was designed without a control group; therefore, we were not able to compare the results from this regimen. In addition, headache, constipation, fatigue, and dizziness are the most common side effects of palonosetron; however, both side effects are also expected postoperative symptoms after craniotomies (18).

## Conclusion

The primary objective of this study was to evaluate the efficacy of triple therapy with palonosetron, dexamethasone, and promethazine for the prevention of PONV in subjects undergoing craniotomies. Our results showed a PONV incidence of 30% and an accumulative incidence of nausea and vomiting of 3 (7.5%). In addition, our data had shown the incidence of a change in QTc interval larger than 60 ms 24 h after surgery was 2.8% (*n* = 1). In conclusion, this study had demonstrated that the prophylactic regimen used could be an adequate regimen to prevent PONV in subjects undergoing craniotomies under general anesthesia. However, since prolongation of QTc interval was not our primary objective, and we did not have a control group; more studies with a proper power analysis and combined therapy should be performed to demonstrate the efficacy of this regimen and that palonosetron is a low risk for QTc prolongation.

## Author Contributions

SB and EP designed the study, developed the methodology, and wrote the manuscript. AU and MA developed the methodology, collected the data, performed the analysis, and wrote the manuscript. GC and VY performed the analysis and wrote the manuscript.

## Conflict of Interest Statement

The authors declare that the research was conducted in the absence of any commercial or financial relationships that could be construed as a potential conflict of interest.
